# Association between dietary inflammatory index and anemia in US adults

**DOI:** 10.3389/fnut.2023.1310345

**Published:** 2024-01-10

**Authors:** Huimiao Ma, Wenqi Deng, Haiyan Chen, Xiaoqing Ding

**Affiliations:** ^1^Department of Hematology, Dongfang Hospital Affiliated to Beijing University of Chinese Medicine, Beijing, China; ^2^Beijing University of Chinese Medicine, Beijing, China

**Keywords:** dietary inflammatory index, anemia, NHANES, cross-sectional study, adult

## Abstract

**Background and aims:**

Anemia is a widespread global health concern, and recent research has unveiled a link between anemia and inflammation. The Dietary Inflammation Index (DII) is a novel tool used to assess the overall inflammatory potential of an individual’s diet. However, until now, there have been no studies demonstrating a connection between DII and anemia. This study aimed to explore the relationship between DII and the risk of anemia among Americans, as well as to examine the influence of other risk factors on this association.

**Methods:**

Data from 32,244 patients were collected from the National Health and Nutrition Examination Survey (NHANES) database spanning from 1999 to 2018. Using multivariable logistic regression, we examined the correlation between DII and anemia. Subgroup analyses and smoothed curve analyses were conducted to further investigate the association between DII and anemia.

**Results:**

The analysis revealed a significant positive association between higher DII scores and increased anemia risk in the American population (Odds Ratio [OR] = 1.06, 95% Confidence Interval [CI] = 1.03 to 1.09, *p* < 0.0001). This association remained consistent in subgroup analyses, encompassing various age groups, distinct Body Mass Index (BMI) categories, varying diabetes mellitus statuses, histories of hypertension, females, individuals with a RIP <3.5, and Non-Hispanic Black individuals. Notably, the association was particularly significant among non-smokers. Smoothed curve fitting analysis demonstrated a linear relationship between DII and the prevalence of anemia.

**Conclusion:**

Our findings underscore a positive correlation between the inflammatory potential of one’s diet and the risk of anemia, especially when coupled with other risk factors. Consequently, reducing the consumption of pro-inflammatory foods may serve as one of the effective measures against the development of anemia. Given the variations in gender, age, BMI, and chronic diseases observed in our study, tailored policies could better cater to the specific needs of diverse populations.

## 1 Introduction

Anemia is characterized by a condition in which the concentration of hemoglobin or red blood cells in the peripheral blood falls below the normal range, leading to the development of various symptoms. This condition represents a significant global public health challenge, with an estimated 32.9% of the world’s population being affected by anemia (1). Notably, anemia is associated with adverse pregnancy outcomes in women (2), serves as an independent prognostic factor for mortality among congestive heart failure patients (3), and contributes to weakness and fatigue, impacting the productivity of adults (4). The prevalence of anemia among the older adult is steadily increasing each year (5) making it a critical concern with far-reaching implications for both human health and socioeconomic development. Therefore, addressing strategies to reduce its prevalence is of paramount importance.

In the context of anemia, inflammation assumes a pivotal role, and anemia of inflammation (AI) stands as the second most common cause of anemia, following iron deficiency anemia (IDA) (6). Research has found that the impact of inflammation on iron homeostasis and the shortening of red blood cell lifespan may exacerbate anemia by inhibiting the differentiation of the erythrocyte lineage (7, 8). Remarkably, dietary components can exert a direct influence on inflammation. Healthy diets, rich in vegetables, whole grains, and fruits, are associated with reduced levels of inflammatory mediators (9); Conversely, Western-style, high-calorie diets coupled with unhealthy lifestyles can induce chronic metabolic inflammation (10). Therefore, quantifying the overall inflammatory potential of an individual’s diet could offer valuable insights for tailoring disease-specific dietary recommendations.

DII is a novel tool used to assess the overall inflammatory potential of an individual’s diet. Elevated DII scores correspond to stronger pro-inflammatory effects, while lower scores indicate more potent anti-inflammatory effects (11). Previous studies have provided initial evidence of associations between DII and an increased risk of number of chronic diseases such as cancer (12), diabetes and cardiovascular risk (13). Furthermore, In recent years, it has become increasingly evident that the spectrum of diseases in which inflammation contributes to anemia has expanded (8). Quantifying the link between dietary inflammatory potential and anemia is crucial for effective prevention and management. Surprisingly, prior research has not delved into this specific relationship. To address this knowledge gap, our study aimed to investigate the association between DII and anemia, making use of publicly accessible data from the National Health and Nutrition Examination Survey (NHANES).

## 2 Materials and methods

### 2.1 Study population and design

**Figure 1:**
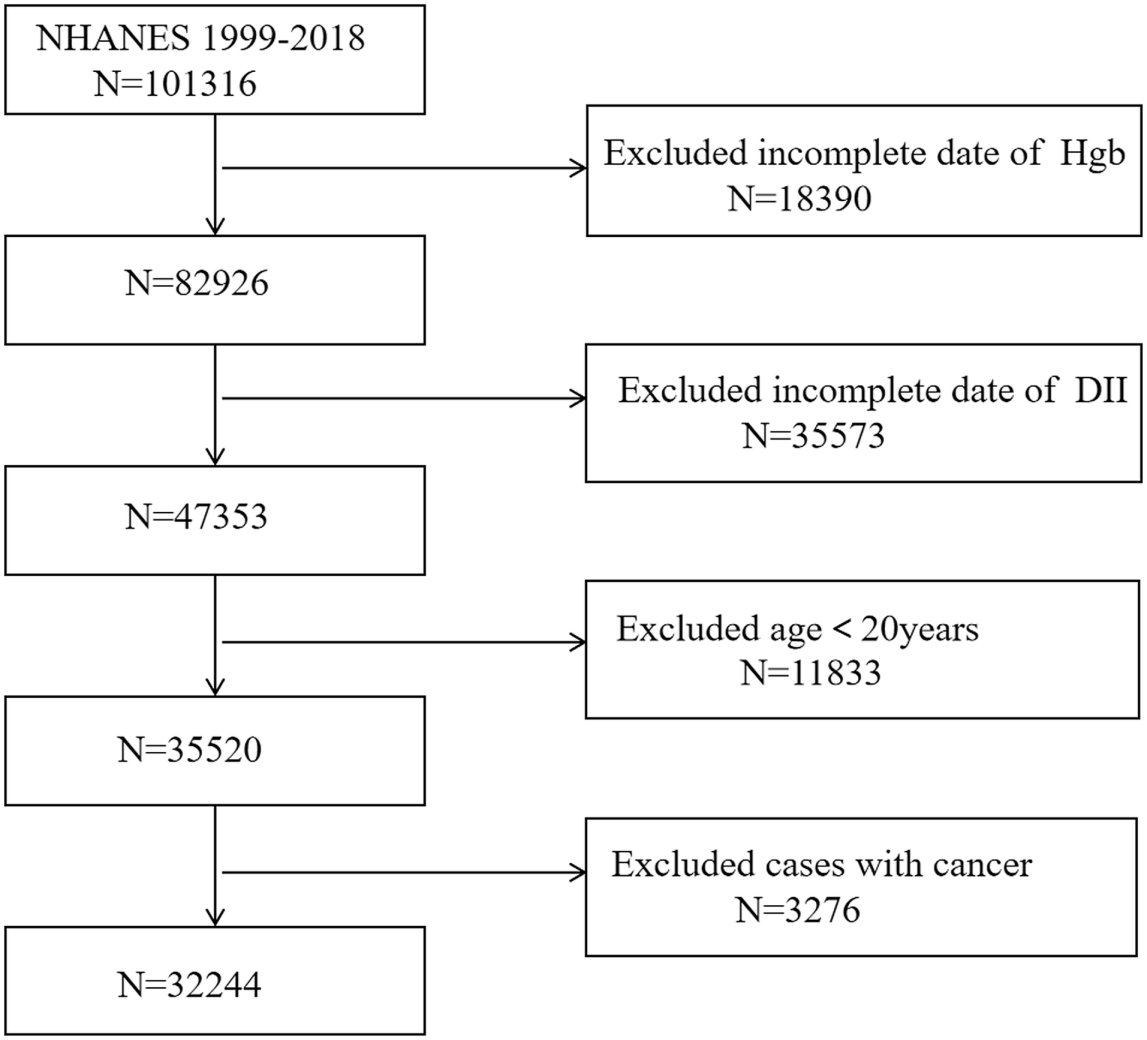
Flow chart of participants selection. NHANES, National Health and Nutrition Examination Survey; DII, dietary inflammatory index.

NHANES is a continuous cross-sectional survey conducted biennially to assess the dietary and health status of the civilian noninstitutionalized U.S. population and is a research project conducted by the National Center for Health Statistics at the Centers for Disease Control and Prevention (14, 15). NHANES utilizes a “stratified multistage probability sampling” approach to ensure a representative sample. In this study, 10 consecutive NHANES cycles between 1999/2000 and 2017/2018 were included. Exclusion criteria for participants in this study were (1) age < 20 years, (2) cancer, and (3) lack of complete data on HBG and DII. Our final analysis included 32,244 eligible participants aged ≥20 years (Figure 1). This study received approval from the Ethical Review Board of the National Center for Health Statistics. All participants provided written informed consent.

### 2.2 Assessment of anemia

Anemia was defined in accordance with the World Health Organization (WHO) criteria (16), with Hgb levels below 12 g/dL for women and below 13 g/dL for men.

### 2.3 Dietary inflammatory index

The DII is now a widely recognized parameter for assessing overall dietary inflammation, and its structural validity and calculation methodology have been published (11). Refer to Supplementary Table S1 for the food parameter-specific overall inflammatory effect score. Dietary data for this study were collected from 24-h dietary recall interviews conducted at a mobile health center as part of the NHANES survey. We computed DII scores based on the 24-h dietary data. First, we calculated various dietary parameters and their respective z-scores for each participant. Then, the values were then converted to median percentiles. For each median percentile, we calculated a standardized overall inflammatory impact score, considering a range of dietary factors. By summing the DII scores for each participant, we obtained an “overall DII score” that reflects the individual’s dietary inflammatory potential. The dietary parameters included in this study encompassed a wide range of factors, including energy, protein, total fat, fiber, carbohydrate, cholesterol, alcohol, vitamins (B12, B6, A, C, E), β-carotene, caffeine, monounsaturated fatty acids (MUFA), n-3 fatty acids, folic acid, iron (Fe), magnesium (Mg), niacin, riboflavin, polyunsaturated fatty acids (PUFA), saturated fat, selenium (Se), thiamin, and zinc (Zn). Participants were categorized into quartiles based on their DII values. This comprehensive approach allowed us to assess the relationship between dietary inflammatory potential and anemia prevalence.

### 2.4 Covariates

In our study, we analyzed several relevant variables, including: Age, Gender, Race (Mexican American, Other Hispanic, Non-Hispanic White, Other Race, Non-Hispanic Black), Academic level (Less Than high school, High School or Equivalent, College or above), Marital Status (Married, Separated, Never married), Ratio of family income to poverty (RIP)(<1.3, 1.3–3.5, >3.5), drinking (alcohol consumption was obtained through the question: “Had at least 12 alcohol drinks in 1 year?”), Smoking (smoking status was obtained through the question: “Smoked at least 100 cigarettes in life?”), Body Mass Index (BMI) (<25, 25 ≤ BMI < 30, ≥30), White Blood Cell, Red Blood Cell, Platelet Count, Hypertension, High Cholesterol, Diabetes, Congestive Heart Failure, Coronary Heart Disease. Detailed information about the measurement processes for these variables is publicly available at www.cdc.gov/nchs/nhanes/.

### 2.5 Statistical analysis

All data were analyzed using R Statistics (version 4.2.0) and Empower Stats software.^1^ Data were weighted and analyzed following the available NHANES guidelines. Normally distributed continuous variables are presented as mean ± standard deviation, while categorical variables are expressed as frequencies or percentages. Statistical methods employed in this study included logistic regression to examine the impact of exposure factors on the disease. To account for the influence of other factors on anemia and to isolate the independent effect of DII on anemia, we conducted multiple regression analysis in three distinct models. In Model 1, no covariates were adjusted. Model 2 incorporated adjustments for gender, age, and race. Model 3 included adjustments for all covariates. Subgroup analyses were also carried out. As DII was considered a continuous variable, we explored potential nonlinear relationships using smooth curve fitting and generalized additive modeling (GAM). A significance level of *p* < 0.05 was employed to determine statistical significance in all analyses.

## 3 Results

### 3.1 Baseline characteristics

A total of 32,244 participants were included in the study, with a mean age of 48.20 ± 17.78 years, consisting of 48.53% males and 51.47% females. Among the participants, 8.85% had anemia.

Table 1 presents the clinical characteristics of individuals with anemia. In comparison to the non-anemic group, participants with anemia exhibited significantly higher DII values (1.30 ± 1.83 vs. 1.83 ± 1.75, *p* < 0.001), were older (45.07 ± 16.07 vs. 49.27 ± 18.28, *p* < 0.001), and had a higher mean BMI (28.64 ± 6.56 vs. 29.69 ± 7.75 kg/m2, *p* < 0.001). Additionally, anemic participants had lower levels of education and annual household income. There were also differences in the racial composition between the two groups. Furthermore, the prevalence of hypertension, diabetes, heart failure, and coronary heart disease was higher among individuals in the anemia group compared to the non-anemic group.

**Table 1:** Weighted characteristics of the study population based on anemia.

	Non-anemia	Anemia	*p*-value
DII	1.30 ± 1.83	1.83 ± 1.75	<0.0001
Age	45.07 ± 16.07	49.27 ± 18.28	<0.0001
Gender (%)			<0.0001
Male	50.65	25.03	
Female	49.35	74.97	
Race (%)			<0.0001
Mexican American	8.54	9.36	
Other Hispanic	5.73	7.12	
Non-Hispanic White	69.77	44.86	
Non-Hispanic Black	9.51	32.21	
Other Race	6.45	6.45	
Academic level (%)			<0.0001
Less than high school	16.94	24.61	
High School or Equivalent	25.02	25.24	
College or above	58.04	50.16	
Marital status (%)			<0.0001
Married	71.10	72.61	
Separated	2.47	4.36	
Never married	26.43	23.03	
RIP (%)			<0.0001
<1.3	19.76	28.74	
1.3–3.5	36.25	41.62	
>3.5	43.99	29.64	
Mean ± SD	3.06 ± 1.62	2.53 ± 1.60	
Drinking (%)			<0.0001
No	14.09	24.00	
Yes	46.14	36.35	
Missing	39.78	39.66	
Smoking (%)			<0.0001
No	53.15	63.59	
Yes	46.85	36.41	
BMI (kg/m^2^) (%)			<0.0001
<25	31.50	31.37	
25 ≤ BMI < 30	33.71	27.92	
≥30	34.79	40.70	
Mean ± SD	28.64 ± 6.56	29.69 ± 7.75	
White blood cell (10^3^ cells/uL)	7.31 ± 2.16	7.15 ± 2.50	0.0024
Red blood cell (10^6^ cells/uL)	4.78 ± 0.46	4.12 ± 0.52	<0.0001
Platelet (10^3^ cells/uL)	258.21 ± 63.98	280.53 ± 91.69	<0.0001
CRP (mg/dL)	0.39 ± 0.72	0.68 ± 1.33	<0.0001
Hypertension (%)			<0.0001
NO	72.92	61.23	
YES	27.08	38.77	
High cholesterol (%)			0.0026
NO	48.77	52.08	
YES	28.10	28.02	
Missing	23.13	19.91	
Diabetes (%)			<0.0001
NO	92.72	82.30	
YES	7.28	17.70	
Congestive heart failure (%)			<0.0001
NO	98.37	94.60	
YES	1.63	5.40	
Coronary heart disease (%)			<0.0001
NO	97.19	93.53	
YES	2.81	6.47	

Mean ± SD for continuous variables; % for categorical variables; BMI, body mass index; DII, dietary inflammatory index; RIP, ratio of family income to poverty; CRP, C-reactive protein.

Table 2 displays the characteristics of the participants categorized by DII quartiles. The mean DII was 1.45 ± 1.82, with quartiles 1 to 4 having DII ranges of −5.28 to −0.20, 0.20 to 1.67, 1.67 to 2.87, and 2.87 to 5.79, respectively. Significant differences were observed among different quartiles of DII for factors such as gender, race, academic level, marital status, RIP, drinking habits, smoking habits, BMI, white blood cell count, red blood cell count, platelet count, C-reactive protein (CRP), hypertension, high cholesterol, diabetes, congestive heart failure, and coronary heart disease. Moreover, the proportion of individuals with anemia increased with higher DII quartiles (Quartile 1: 4%, Quartile 2: 4.97%, Quartile 3: 6.23%, Quartile 4: 8.30%; *p* < 0.0001). Similarly, the average hemoglobin (Hgb) level for all participants was 14.18 ± 1.56. The average hemoglobin level for the highest quartile was 14.08 ± 1.54, while for the lowest quartile, it was 14.61 ± 1.39 (*p*<0.05). Importantly, white blood cell counts and C-reactive protein (CRP) levels also increased with the rising quartiles of DII (*p*<0.05).

**Table 2:** Weighted characteristics of the study population based on DII quartiles.

	DII Quartiles				
	Quartile1 −5.28–0.20	Quartile2 0.20–1.67	Quartile3 1.67–2.87	Quartile4 2.87–5.79	*p*-value
Age	45.37 ± 15.52	45.13 ± 15.82	44.89 ± 16.53	45.90 ± 17.16	0.0010
Gender (%)					<0.0001
Male	62.01	52.13	44.54	35.63	
Female	37.99	47.87	55.46	64.37	
Race (%)					<0.0001
Mexican American	8.65	9.42	8.45	7.71	
Other Hispanic	5.25	6.44	5.77	5.80	
Non-Hispanic White	71.80	67.52	67.80	65.70	
Non-Hispanic Black	7.31	9.92	11.98	14.74	
Other Race	6.99	6.69	5.99	6.05	
Academic level (%)					<0.0001
Less than high school	12.94	16.66	18.65	22.10	
High School or Equivalent	20.28	24.39	26.22	30.10	
College or above	66.78	58.95	55.14	47.80	
Marital status (%)					<0.0001
Married	73.10	72.53	70.15	68.59	
Separated	1.85	2.50	2.80	3.28	
Never married	25.05	24.97	27.05	28.13	
RIP (%)					<0.0001
<1.3	15.39	18.36	21.31	27.06	
1.3–3.5	32.06	36.31	38.66	39.89	
>3.5	52.56	45.33	40.03	33.05	
Mean ± SD	3.36 ± 1.61	3.13 ± 1.61	2.92 ± 1.60	2.64 ± 1.60	
Drinking (%)					<0.0001
No	10.70	13.12	15.33	20.35	
Yes	46.13	44.15	45.89	46.15	
Missing	43.17	42.73	38.79	33.50	
Smoking (%)					<0.0001
No	56.29	55.27	52.18	50.74	
Yes	43.71	44.73	47.82	49.26	
BMI (kg/m^2^) (%)					<0.0001
<25	34.64	32.12	29.82	28.82	
25 ≤ BMI < 30	34.94	34.59	33.10	30.47	
≥30	30.43	33.28	37.08	40.71	
Mean ± SD	28.00 ± 6.23	28.54 ± 6.46	28.98 ± 6.75	29.39 ± 7.09	
White blood cell (10^3^ cells/uL)	7.01 ± 1.96	7.26 ± 2.22	7.44 ± 2.12	7.54 ± 2.40	<0.0001
Red blood cell (10^6^ cells/uL)	4.79 ± 0.48	4.76 ± 0.48	4.73 ± 0.49	4.68 ± 0.49	<0.0001
Hgb (g/dL)	14.61 ± 1.39	14.47 ± 1.45	14.33 ± 1.50	14.08 ± 1.54	<0.0001
Platelet (10^3^ cells/uL)	250.25 ± 61.43	257.70 ± 63.89	263.64 ± 66.74	268.01 ± 71.49	<0.0001
CRP (mg/dL)	0.32 ± 0.74	0.38 ± 0.67	0.43 ± 0.74	0.49 ± 0.91	<0.0001
Hypertension (%)					<0.0001
NO	74.46	73.57	71.92	68.49	
YES	25.54	26.43	28.08	31.51	
High cholesterol (%)					<0.0001
NO	50.63	49.58	47.91	47.41	
YES	29.07	27.42	27.78	28.04	
Missing	20.30	23.01	24.32	24.54	
Diabetes (%)					<0.0001
NO	93.15	92.59	91.85	90.64	
YES	6.85	7.41	8.15	9.36	
Congestive heart failure (%)					<0.0001
NO	98.69	98.42	97.82	97.58	
YES	1.31	1.58	2.18	2.42	
Coronary heart disease (%)					0.0379
NO	97.09	97.36	96.74	96.66	
YES	2.91	2.64	3.26	3.34	
Anemia (%)					<0.0001
NO	96.00	95.03	93.77	91.70	
YES	4.00	4.97	6.23	8.30	

Mean ± SD for continuous variables; % for categorical variables; BMI, body mass index; RIP, ratio of family income to poverty; Hgb, hemoglobin; DII, dietary inflammatory index; CRP, C-reactive protein.

### 3.2 Association between DII and anemia

**Figure 2:**
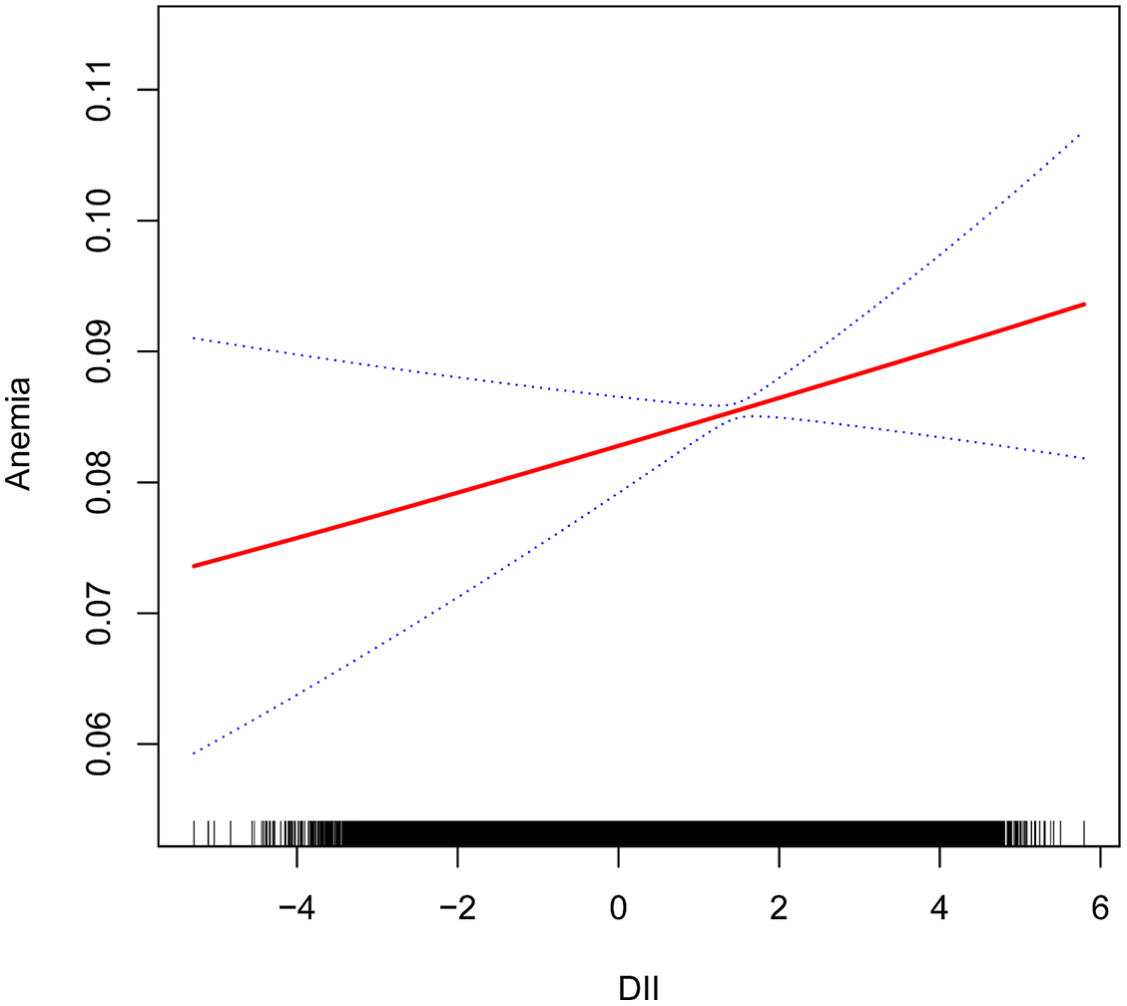
Relationship between dietary inflammatory index and anemia. The vertical axis represents log OR of anemia. DII, dietary inflammatory index.

We performed a multivariable logistic regression analysis to investigate the association between DII and anemia. Both unadjusted and adjusted models are detailed in Table 3. When compared to the lowest quartile of DII, a higher prevalence of anemia was observed in the fourth quartile in Model 1 (OR = 1.90, 95% CI = 1.69–2.12, *p* < 0.01), Model 2 (OR = 1.36, 95% CI = 1.21–1.53, *p* < 0.01), and Model 3 (OR = 1.35, 95% CI = 1.17–1.57, *p* < 0.01). Across all models, there was a significant association between higher DII levels and an increased prevalence of anemia (*p* for trend <0.01). Furthermore, even after adjusting for all covariate confounders, smoothed curve fitting analysis (Figure 2) revealed a linear relationship between DII and anemia.

**Table 3:** The odds ratio for the relationship between DII and anemia.

	MODEL 1 OR (95%CI) *p*-value	MODEL 2 OR (95%CI) *p*-value	MODEL 3 OR (95%CI) *p*-value
DII	1.15 (1.12, 1.17) <0.0001	1.07 (1.04, 1.09) <0.0001	1.06 (1.03, 1.09) <0.0001
DII Quintiles			
Quartile 1	Reference	Reference	Reference
Quartile 2	1.27 (1.12, 1.43) 0.0001	1.12 (0.99, 1.27) 0.0620	1.17 (1.01, 1.36) 0.0422
Quartile 3	1.51 (1.34, 1.70) <0.0001	1.23 (1.09, 1.38) 0.0008	1.20 (1.04, 1.40) 0.0158
Quartile 4	1.90 (1.69, 2.12) <0.0001	1.36 (1.21, 1.53) <0.0001	1.35 (1.17, 1.57) <0.0001
*p* for trend	<0.0001	<0.0001	<0.0001

MODEL 1: No covariates were adjusted. MODEL 2: Age, Gender, Race were adjusted. MODEL 3: Age, Gender, Race, Academic level, Marital status, RIP, drinking, Smoking, BMI, White blood cell, Red blood cell, Platelet, Hypertension, High cholesterol, Diabetes, Congestive heart failure, Coronary heart disease were adjusted. 95% CI, 95% confidence interval; OR, odds ratio; DII, dietary inflammatory index.

### 3.3 Subgroup analysis

Subgroup analyses were conducted to further explore the relationship between DII and anemia in different populations, as shown in Table 4. In subgroup analyses according to Age, Gender, Race, RIP, Drinking, Smoking, BMI, Diabetes and Hypertension. The results showed a positive association between DII and anemia in different age, BMI, alcohol, diabetes, and hypertension groups. Specifically, in individuals aged 60 or younger, the prevalence of anemia increased by 4% with each unit increase in DII, while in those over 60, the prevalence increased by 9% per unit increase in DII. For non-diabetic patients, each unit increase in DII was associated with a 5% increase in the prevalence of developing anemia, compared to an 11% increase in diabetic patients.

**Table 4:** Subgroup analysis of the association between dietary inflammatory index with anemia.

	OR (95%CI) *p*-value	*p* for interaction
Age		0.1442
≤60	1.04 (1.00, 1.08) 0.0395	
>60	1.09 (1.03, 1.14) 0.0009	
Gender		0.3033
Male	1.03 (0.98, 1.09) 0.2443	
Female	1.07 (1.03, 1.10) 0.0003	
Race		0.4108
Mexican American	1.06 (0.99, 1.14) 0.1023	
Other Hispanic	1.10 (0.98, 1.23) 0.0999	
Non-Hispanic White	1.01 (0.95, 1.07) 0.7373	
Non-Hispanic Black	1.07 (1.02, 1.12) 0.0069	
Other Race	1.10 (1.00, 1.21) 0.0472	
RIP		0.7894
<1.3	1.06 (1.01, 1.11) 0.0285	
1.3–3.5	1.07 (1.02, 1.12) 0.0039	
>3.5	1.04 (0.99, 1.10) 0.1315	
BMI		0.9939
<25	1.06 (1.01, 1.11) 0.0284	
25 ≤ BMI < 30	1.06 (1.01, 1.12) 0.0311	
≥30	1.06 (1.01, 1.11) 0.0117	
Drink		0.8934
NO	1.06 (1.00, 1.12) 0.0462	
Yes	1.05 (1.00, 1.10) 0.0338	
Smoking		0.0110
NO	1.09 (1.05, 1.13) <0.0001	
YES	1.00 (0.96, 1.05) 0.9011	
Diabetes		0.1237
NO	1.05 (1.02, 1.08) 0.0033	
YES	1.11 (1.04, 1.20) 0.0028	
Hypertension		0.1647
NO	1.04 (1.01, 1.08) 0.0233	
YES	1.09 (1.04, 1.14) 0.0005	

Adjusted for all covariates except effect modifier.

**Figure 3:**
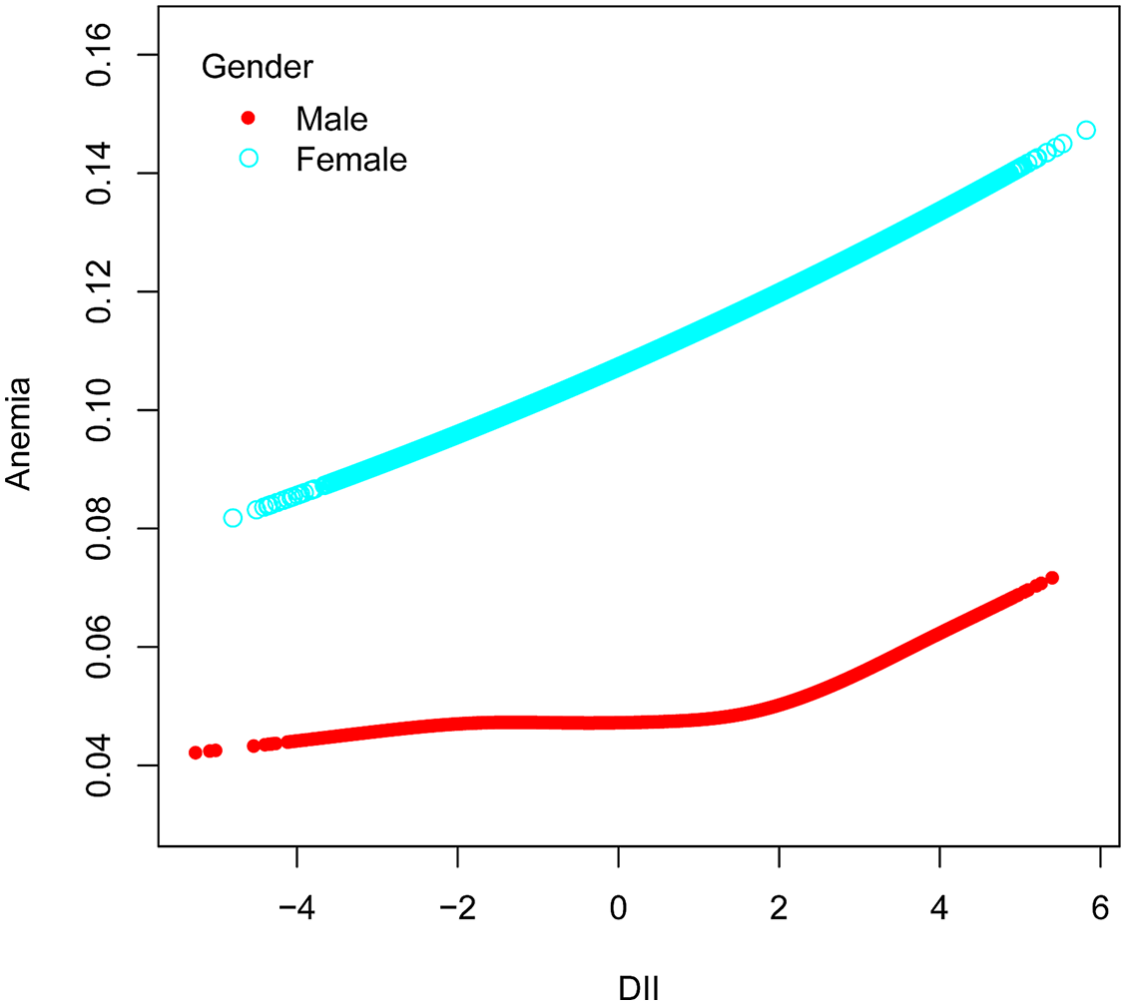
Association between anemia and DII stratified by gender. The vertical axis represents log OR of anemia. DII, dietary inflammatory index.

**Figure 4:**
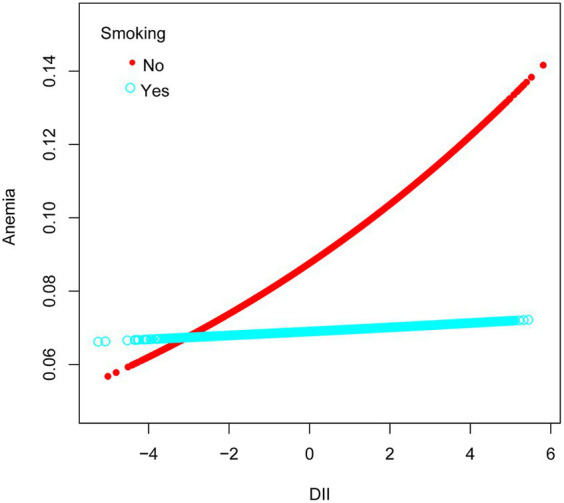
Association between anemia and DII stratified by smoking, respectively. The vertical axis represents log OR of anemia. DII, dietary inflammatory index.

Among ethnic groups, there was no significant association between DII and anemia in Mexican American, Other Hispanic, and Non-Hispanic White groups. However, in the Non-Hispanic Black group, a strong correlation was observed between DII and anemia (OR = 1.07, 95% CI 1.02–1.12, *p* = 0.0069). Subsequently, we found that when RIP <1.3, DII had an effect on the prevalence of developing anemia, with each unit increase in DII increased the prevalence of developing anemia by 6%, and when the RIP was 1.3–3.5, the prevalence of developing anemia increased by 7% for each additional unit of DII. There was also a correlation between DII and anemia when the gender was female (OR = 1.07, 95% CI 1.03–1.10, *p* = 0.0003). In the male population, no significant association was observed between DII and anemia (Figure 3). Simultaneously, in the adjusted model, we observed a significant interaction between smoking and DII (*p* for interaction <0.05). A positive correlation between DII and anemia was evident in non-smokers, who comprised 60.5% women, (OR = 1.09, 95% CI 1.05–1.13, *p* < 0.0001), while no such correlation was found in smokers (OR = 1.00, 95% CI 0.96–1.05, *p* = 0.9011). Therefore, this nonlinear relationship was described by smooth curve fitting and generalized additive modeling (Figure 4).

## 4 Discussion

In this cross-sectional study involving 32,244 participants, the results consistently demonstrated a positive association between anemia and DII, both when adjusted and unadjusted for covariates. These findings suggest that an elevation in DII may indeed contribute to a heightened prevalence of anemia. In light of the findings presented, we propose that integrating dietary modifications aimed at lowering the DII may serve as a complementary strategy in the multifactorial management of anemia.

Recent decades of research have increasingly highlighted a critical link: the connection between dietary patterns, types of food, inflammation, and the risk of diseases (17, 18). In this context, the Dietary Inflammation Index emerges as a valuable tool for understanding the interplay between diet and inflammation. A systematic review and meta-analysis revealed that diets primarily based on plant foods are associated with reduced levels of serum CRP, an indicator of inflammation, and total white blood cell (WBC) counts, markers of innate immunity (19). Moreover, randomized controlled trials have shown that vegetarian diets result in lower DII scores compared to meat-based diets (20). Correspondingly, in our study population, higher DII scores were associated with increased WBC and CRP levels. These results underscore the importance of dietary choices in controlling inflammation and reducing the risk of related diseases.

Anemia represents a widespread global health concern, and recent studies have unveiled a significant link between anemia and inflammation (21, 22). In animal models of AI and in patients with inflammatory diseases, elevated hepcidin levels are linked to decreased ferroportin expression in duodenal enterocytes and macrophages, resulting in impaired dietary iron absorption (23). Furthermore, even low-grade inflammation can hinder intestinal iron absorption (24), which consequently leads to a reduced iron supply for erythropoiesis (25), and thus negatively impacting anemia. Inflammatory cytokines additionally play a role in reducing the lifespan of red blood cells, possibly by activating macrophages (6). Given this backdrop, dietary patterns, especially pro-inflammatory diets, play a crucial role. Pro-inflammatory diets, by increasing the body’s inflammatory responses, may heighten the risk of anemia. A prospective cohort study (26) on anemia in pregnant women has shown that higher pro-inflammatory diet scores correlate with lower hemoglobin (HGB) levels in expectant mothers. Including an anti-inflammatory diet may help prevent maternal anemia in women with gestational diabetes. Thus, it appears that focusing on dietary factors, especially in reducing pro-inflammatory diets, may play a significant role in managing anemia. It is important to note that while adhering to a vegetarian diet may help reduce the DII, individuals prone to anemia need to carefully manage their diet and supplements to maintain optimal health. High levels of phytates in grains and legumes can interfere with the absorption of essential minerals like calcium, zinc, iron, iodine, and magnesium (27). Consequently, diets rich in these foods may lead to mineral deficiencies (28). Although plant foods are high in iron, studies show that strict vegetarians are at a higher risk of iron deficiency (29). Plant-based iron, being non-heme, is less bioavailable compared to the heme iron found in animal products (28, 30). Therefore, a practical approach could be the moderate inclusion of nutrient-rich animal-sourced foods for a more balanced dietary intake.

Our results highlights that factors such as gender, age, ethnicity, RIP, body mass index, and diabetes, exhibit similar directional effects on the relationship between the prevalence of anemia and DII. The findings related to age, ethnicity, and economic status align with the results obtained from the world population Anemia risk survey (16). Subgroup analysis revealed a stronger DII impact on anemia in individuals over 60, females, non-smokers, those of Non-Hispanic Black ethnicity, economically disadvantaged groups, and patients with hypertension, diabetes mellitus, or heart failure. The overlapping effects of pro-inflammatory foods and these risk factors on anemia prevalence are noteworthy.

Numerous studies have revealed disparities in anemia across different regions and ethnic groups (1, 16), which may be closely related to the dietary cultures of different ethnicities. For instance, research reports indicate that the intake of fruits and vegetables is generally lower among the Black/African American population (31). Furthermore, compared to white individuals with anemia, Black individuals exhibit a higher incidence rate of inflammation related to anemia (32). Our cross-sectional study results also emphasize the importance of the relationship between inflammatory diets and the prevalence of anemia. How to integrate traditional dietary habits of different ethnic groups with anti-inflammatory principles requires further exploration. Understanding the dietary differences between ethnic groups and their impact on food choices is crucial in formulating effective and culturally adaptive public health strategies.

Notably, anemia tends to be more prevalent in women compared to men (16), partly due to factors like menstrual cycles, pregnancy, and higher susceptibility to iron deficiency in high-DII environments (33). The interaction between female physiological differences, including sex hormone variations, and DII is complex and merits deeper exploration. For instance, initial studies suggest a link between DII and sex hormones in female adolescents (34) and the role of estrogen in inflammatory responses has been recognized (35). These findings indicate that estrogen and DII may jointly influence anemia development in women. Furthermore, gender-specific dietary habits (36), including food choices and intake, could contribute to the varied impact of DII on anemia between men and women.

Smoking, a key factor linked to inflammation and various diseases (37), necessitates detailed examination for its potential role in modulating the relationship between dietary inflammatory indices and anemia. The presence of harmful chemicals like tar, nicotine, and carbon monoxide in tobacco smoke could impact the immune system, possibly suppressing or altering its inflammatory response (38–40). While smoking itself triggers inflammation, it may also weaken the immune response in high-inflammatory settings, potentially reducing susceptibility to the effects of a pro-inflammatory diet. Compared to non-smokers, smokers have been found to consume fewer fruits and vegetables while having a higher intake of fats and alcohol (41, 42). These dietary discrepancies could potentially affect the relationship between the DII and anemia. The particular effects of these dietary habits on DII in smokers, however, remain under-researched. Additional studies are needed to investigate the influence of smoking on DII and iron status. Consequently, comprehending the complex role of smoking in this relationship is vital, necessitating further research to decode the underlying mechanisms.

Age, chronic diseases, and dietary inflammatory indices are interconnected in anemia development. Diseases often associated with anemia, like myelodysplastic syndromes (MDS), chronic kidney disease (CKD), and gastrointestinal (GI) conditions, are prevalent in older individuals (43). A pro-inflammatory state, common in the older adult, is increasingly recognized as a contributor to anemia (5). Additionally, anemia is frequently associated with a higher BMI, which not only indicates elevated inflammatory cytokine levels (44) but also tends to correlate with diets high in calories and fats, yet low in fiber (45). This dietary pattern raises DII, and, when combined with the heightened risk of metabolic and cardiorespiratory diseases associated with high BMI (46), can intensify anemia through inflammation. Furthermore, recent evidence indicates a potential link between pro-inflammatory diets and an increased incidence of chronic diseases such as diabetes (47) and hypertension (48). Our stratified analysis indicating a heightened risk of anemia in those with these chronic conditions. Therefore, it appears that adopting a low-inflammatory dietary pattern might be beneficial in potentially reducing factors associated with anemia. This approach aims to address not only the direct dietary contributors to anemia but also the chronic conditions that may exacerbate its occurrence. However, it is important to note that this hypothesis requires further investigation. In conclusion, effective management of anemia necessitates acknowledging and addressing these interconnected factors. A comprehensive approach, tailored to individual needs and considering the intricate interplays among DII, gender-specific factors, smoking, age, chronic diseases, and BMI, is essential for the precise prevention and treatment of anemia.

However, it’s essential to acknowledge certain limitations in the current study that require consideration. Firstly, the cross-sectional design of this study inherently limits its ability to establish causality. Secondly, it is important to note that our study faced limitations in directly linking DII with specific types of anemia, primarily due to sample size constraints regarding laboratory tests related to anemia (e.g., iron deficiency, vitamin deficiencies, etc.) within the database. This limitation may have influenced the precision of our results. Thirdly, the study’s results may not be generalizable to younger patients, as some participants under the age of 20 were not included in the analysis. Finally, the anemia data used in this study were based on laboratory diagnoses and may not encompass patients with a historical record of anemia, potentially leading to incomplete results.

## 5 Conclusion

Our study reveals an association between an inflammatory diet and a heightened prevalence of anemia, with a more pronounced effect observed in women, older individuals, those with higher BMI, non-smokers, Non-Hispanic Black individuals, and those with less favorable family conditions. These findings offer valuable insights for public health authorities and healthcare providers, enabling the development of more precise strategies for anemia screening and prevention. The potential role of dietary modification, specifically reducing inflammatory dietary patterns, warrants further investigation. While these preliminary observations suggest a direction for public health interventions, robust clinical trials are required to establish causative links and to develop effective, targeted dietary guidelines for anemia prevention, particularly in demographically diverse populations.

## Data availability statement

Publicly available datasets were analyzed in this study. This data can be found at: https://www.cdc.gov/nchs/nhanes/.

## Ethics statement

The studies involving humans were approved by National Center for Health Statistics. The studies were conducted in accordance with the local legislation and institutional requirements. The participants provided their written informed consent to participate in this study.

## Author contributions

HM: Conceptualization, Formal analysis, Methodology, Supervision, Writing – original draft. WD: Data curation, Formal analysis, Software, Supervision, Writing – original draft. HC: Formal Analysis, Software, Writing – review & editing. XD: Conceptualization, Writing – review & editing.

## Footnotes

^1^ http://www.empowerstats.net
