# Supplementary material for: Association between dietary inflammatory index and anemia in US adults
Source: Front Nutr. 2024 Jan 10;10:1310345. doi: 10.3389/fnut.2023.1310345 (PMC10805879; doi:10.3389/fnut.2023.1310345)
Supplement: Supplementary file 1 [file Data_Sheet_1.PDF]

Supplemental Table 1 Overall Inflammatory Effect Score for 26 Specific Food Parameters

Food	Overall inflammatory effect score
Alcohol (g)	−0.278
Vitamin B12 (μg)	0.106
Vitamin B6 (mg)	−0.365
β-Carotene (μg)	−0.584
Caffeine (g)	−0.110
Carbohydrate (g)	0.097
Cholesterol (mg)	0.110
Energy (kcal)	0.180
Total fat (g)	0.298
Fiber (g)	−0.663
Folic acid (μg)	−0.190
Fe (mg)	0.032
Mg (mg)	−0.484
MUFA (g)	−0.009
Niacin (mg)	−0.246
n-3 Fatty acids (g)	−0.436
Protein (g)	0.021
PUFA (g)	−0.337
Riboflavin (mg)	−0.068
Saturated fat (g)	0.373
Se (μg)	−0.191
Thiamin (mg)	−0.098
Vitamin A (RE)	−0.401
Vitamin C (mg)	−0.424
Vitamin E (mg)	−0.419
Zn (mg)	−0.313
